# A Wideband Trapezoidal Cantilever Beam PVEH with a P-SSHI-QVR Circuit for Low-Frequency Applications

**DOI:** 10.3390/mi16121414

**Published:** 2025-12-16

**Authors:** Yan Jin, Boyi Feng, Yubo Jin, Yiwen Lv, Zhifan Zhao, Jiaqi Ju, Zhengguang Shi

**Affiliations:** 1 College of Sciences, Shanghai Institute of Technology, Shanghai 201418, China; 2 School of Art and Design, Shanghai Institute of Technology, Shanghai 201418, China

**Keywords:** wideband, trapezoidal, PVEH, a P-SSHI-QVR circuit, low-frequency

## Abstract

Piezoelectric vibration energy harvesters (PVEHs) have demonstrated their potential for sustainable energy generation from diverse ambient vibrations for low-power devices and systems. However, great challenges remain concerning harvesting more energy from low-frequency input sources and broadband random excitations. In this paper, a novel PVEH featuring a lead zirconate titanate (PZT) hollowed trapezoidal cantilever beam is proposed, simulated, optimized and fabricated to effectively broaden its output bandwidth at low frequency ranges. Under 1 g acceleration, the traditional solid PVEH showed a resonant frequency of 47.80 Hz and a maximum output power density of 14.22 mW/cm^3^, while the proposed PVEH showed two resonant frequencies of 21.30 Hz and 50.40 Hz. Compared to the traditional solid PVEH, the first-order resonant frequency was reduced by 55.44% and the corresponding maximum output power density was 3.3 times higher in the proposed PVEH. Furthermore, a parallel synchronized switch harvesting inductor quadruple voltage rectifier (P-SSHI-QVR) circuit is designed to extract energy from the proposed PVEH. For the proposed PVEH incorporating the P-SSHI-QVR circuit, the maximum stored voltage was 20.49 V at a first-order resonant frequency of 21.30 Hz and 5.68 V at a second-order resonant frequency of 50.40 Hz, with corresponding maximum stored powers of 36.89 μW and 2.97 μW, respectively. This study verified the feasibility of the optimized design through simulation and experimental comparison.

## 1. Introduction

Power supply has become a problem restricting the durability of micro-devices, as traditional chemical batteries are relatively large in size, have a short service life [1], are environmentally unfriendly and need to be replaced regularly [2]. In recent years, with the development of low-power devices and the continuous reduction in power consumption of micro-devices, capturing energy from the environment to power micro-devices has become possible. Vibration energy harvesters, which can transform ambient vibration energy from diverse ambient vibration sources, including human motion, acoustic vibration, machine vibration, and stress into electrical energy, are being used as an alternative to traditional chemical batteries to power low-power wireless sensors and wearable devices, and have attracted increasing research interest in recent years [3].

Diverse transduction mechanisms based on piezoelectric, electromagnetic, electrostatic or hybrid systems [3,4,5,6,7,8] have been utilized to convert vibrations into electrical energy. Among these, piezoelectric vibration energy harvesters (PVEHs) adopting the positive piezoelectric effect of piezoelectric materials to complete the electromechanical conversion process [9,10] have been investigated extensively due to their very simple structures, weatherproof properties, ability to be miniaturized, and high energy densities [11,12]. When an external force is imposed on a piezoelectric material, a relative movement of charges inside the material would be caused to generate polarization, and anisotropic bound charges would be induced on the two surfaces of the piezoelectric element [13], which can be stored or used directly as an electromechanical energy converter or energy harvester [14]. As PVEHs utilize the deformation of the piezoelectric material to generate power, they do not require an independent drive unit and have a simple configuration [10]. Therefore, PVEHs are easily miniaturized, integrated, mass-produced and highly compatible with the MEMS process [15].

The typical structure of PVEHs is based on the cantilever beam structure with one clamped end and one free end attached with a proof mass. The piezoelectric layer, which mainly comprises piezoelectric materials such as piezoelectric single crystals, piezoelectric ceramics, piezoelectric polymers and piezoelectric composite materials [16,17], is deposited on a substrate for the conversion of mechanical energy into electrical energy [18], wherein PZT-based piezoelectric materials have been considered as the most outstanding piezoelectric materials for energy harvesting due to their much higher piezoelectric coefficients and electromechanical coupling coefficient [19,20]. PVEHs can produce significant response amplitude only when the main ambient vibration frequency exactly matches the resonant frequency of the harvester [21]. If the ambient vibration frequency deviates from the resonant frequency by about 2%, the output voltage drops to about 50% of the resonance peak. If the deviation is 5%, there is almost no output [22]. Therefore, it is challenging for the cantilever-type PVEHs to extract energy from the variable environments due to their narrow bandwidth. Most environmental vibrations occur within a low frequency range, typically below 100 Hz [23]. Thus, PVEHs should be designed to efficiently harvest energy from wideband environments, especially those with low frequencies.

In general, for ultra-low-frequency vibration sources (typically below 10 Hz), a single cantilever beam without a proof mass is not sufficient. Additional design strategies are often adopted to further reduce the effective operating frequency band. These include attaching a relatively large tip mass and introducing frequency up-conversion mechanisms (e.g., impact or plucking structures) [9,18]. In this study, the term “low-frequency” is employed to denote vibrations in the range of approximately 20–40 Hz, which are frequently encountered in practical mechanical environments. The proposed hollow trapezoidal cantilever beam is therefore optimised to achieve a relatively low first resonant frequency and a wide bandwidth within this range, while maintaining a compact, proof-mass-free configuration.

To achieve this, mainly two different methods have been proposed by researchers. One is the frequency up-conversion (FUC) technique, which is capable of transforming low-frequency vibrations into high-frequency oscillations [2,9,18,24], and the other one is the cantilever beam structure optimization technique, which can broaden the frequency range and convert high- frequency vibrations to low-frequency ones by integrating multiple cantilever beams into a unified structure, optimizing the size and configuration of the cantilever beams, or adding tip masses [25,26,27,28]. Both techniques, however, still confront certain challenges. For the FUC technique, the ability to conveniently adjust the central working frequency of a wideband energy harvester without necessitating refabrication or causing damage to the original structure is still challenging. As for the cantilever beam structure optimization technique, systematic studies and the development of matching circuits have yet to be explored in detail.

Furthermore, before PVEHs can be utilized as a power source, rectification and voltage stabilization circuits are necessary, as the signals produced by PVEHs are alternating current (AC) voltage signals. The research in reference [29] presents a comparison between the full-bridge rectifier (FBR) circuit and the synchronous switch harvesting inductor (SSHI) circuit. The measurement results indicate that the FBR circuit suffers from a large energy loss due to the inherent capacitor present in the PVEH. In contrast, the SSHI circuit substantially mitigates energy loss during the capacitor charging process by reversing the voltage polarity at the zero-crossing point of the current source.

In this paper, a high-performance PVEH system comprising a hollowed trapezoidal cantilever beam PVEH and a parallel synchronous switch harvesting inductor quadruple voltage rectification (P-SSHI-QVR) circuit is proposed and investigated. The structure of the proposed PVEH is optimized via mechanical analysis and the finite element method (FEM). Subsequently, the prototype system is fabricated and the energy harvesting performance characteristics such as the output voltage and power density are evaluated in detail. For the proposed PVEH incorporating the P-SSHI-QVR circuit, output powers of 36.89 μW and 2.97 μW were achieved at the resonant frequencies of 21.30 Hz and 50.40 Hz, with corresponding maximum storage voltages of 20.49 V and 5.68 V, respectively. This study verified the feasibility of the optimized design through simulation and experimental comparison.

## 2. Design and Simulation

**Figure 1:**
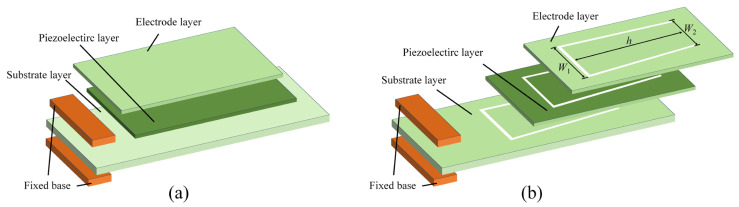
Schematic diagrams of (**a**) the traditional PVEH and (**b**) the proposed trapezoidal cantilever beam PVEH.

The structures and components of a traditional PVEH and the proposed trapezoidal cantilever beam PVEH are shown in Figure 1a and Figure 1b, respectively. As can be seen, for both PVEHs, the left end is the fixed end of the cantilever, which is fastened to the excitation platform by a fixed base, and the right end is the free end, which would shake up and down with the vibration. In this work, a silver layer, a PZT layer, and a copper substrate with optimized sizes [28] are adopted for both PVEHs as the electrode layer, the piezoelectric layer and the substrate layer, respectively. Copper is chosen as the substrate material because it possesses a sufficiently high Young’s modulus (1.12 × 1011 N/m^2^, see Table 1) to provide the required bending stiffness while helping to lower the resonant frequency into the targeted low-frequency range, and its excellent electrical conductivity, solderability and machinability are beneficial for forming the hollowed trapezoidal structure and for reliably connecting the PVEH to the external circuit [28]. Compared to the traditional solid PVEH shown in Figure 1a, the proposed PVEH shown in Figure 1b has a hollowed trapezoidal cantilever beam structure with a bottom width W1 near the fixed end, a bottom width W2 near the free end, and a trapezoidal height h between the two bottoms.

The material types and parameters of PVEHs in the simulation and experiment are shown in Table 1. All finite-element simulations are carried out using COMSOL Multiphysics 6.3 with coupled solid mechanics and electrostatics (piezoelectric) modules. The body loads in the solid mechanics module are simulated for external vibrations, and the entire geometry is discretized using a free tetrahedral mesh with a finer mesh near the fixed end and along the interfaces between the PZT layer and the copper substrate to ensure convergence of the stress and electric-field distributions. In the FEM model, the external vibration is represented as a harmonic base acceleration $a\left(t\right)\;=\;A\;sin(2\pi ft)$ applied in the out-of-plane direction of the cantilever, where $A\;=\;1\;g\approx 9.795{\;m/s}^{2}$ and f is the excitation frequency. The vibration mass-spring-damper base excitation system [28,30] is adopted to describe the energy obtained from the vibration source, wherein the mass of the vibration source is assumed to be much larger than the seismic mass in the generator and the vibration source is assumed to be an infinite power source [31]. On this basis, the detailed dynamic response and output characteristics of the proposed hollowed trapezoidal cantilever beam are obtained directly from the three-dimensional coupled FEM model described below, rather than from a lumped-parameter analytical expression.

**Figure 2:**
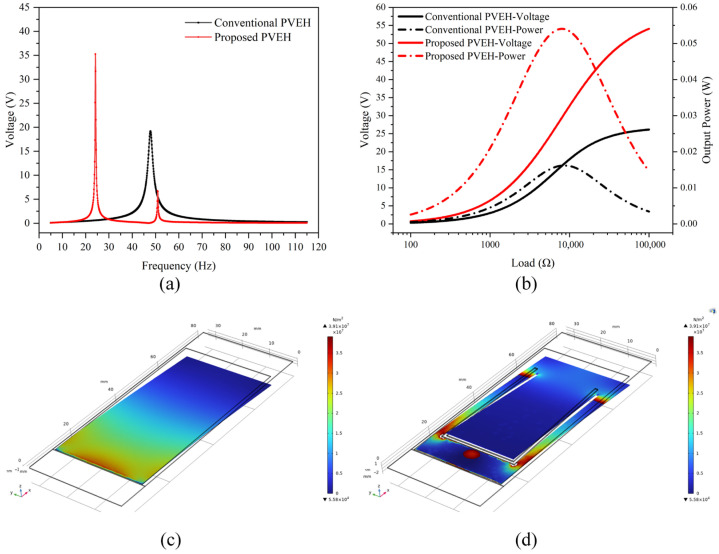
Simulation results of (**a**) output voltages at different frequencies for both PVEHs; (**b**) output voltages (**left** axis) and output power densities (**right** axis) at different loads for both PVEHs; and surface stress distributions of (**c**) the traditional PVEH and (**d**) the proposed PVEH under a harmonic base acceleration of A = 1 g at their first resonant frequencies (47.80 Hz and 24.20 Hz, respectively).

A three-dimensional piezoelectric cantilever beam model with the dimension parameters depicted in Table 2 is built, in which the PZT layer and the copper substrate are modeled as separate solid domains that are mechanically bonded by continuity conditions at their interface, while the thin Ag electrode layer is neglected in the structural model because of its much smaller thickness and stiffness. Coupled analysis of solid mechanics, electrostatics and piezoelectric effects on the PVEHs is then performed, and the open-circuit output voltage shown in Figure 2a is obtained as the potential difference between the upper and lower electrode surfaces at the free end of the cantilever.

In this model, the voltage presented in Figure 2a corresponds to the open-circuit output, i.e., the potential difference between the upper and lower electrode surfaces at the free end of the cantilever when no external load is connected.

To better demonstrate the characteristics of the two types of PVEHs, the acceleration amplitude in the harmonic excitation is fixed at A = 1 g (approximately 9.795 m/s^2^). The output voltages of both the conventional solid PVEH with its dimension parameters detailed in Table 2 and the proposed PVEH featuring an additional hollowed trapezoidal cantilever beam (with dimensions of W1 = 24 mm, W2 = 21 mm, and h = 40 mm) are then calculated by sweeping the excitation frequency f from 5 to 115 Hz with a step size of 0.1 Hz. The results of these FEM calculations are presented in Figure 2a. It should be noted that in these simulations the material properties listed in Table 1 and the geometrical dimensions given in Table 2 and in the optimization study are chosen to be identical to those of the fabricated prototypes, i.e., the conventional solid PVEH (Sample A) and the optimal hollowed trapezoidal PVEH (Sample C with W1 = 24 mm and W2 = 21 mm). As can be observed, within the frequency range of 5 to 115 Hz, the conventional solid PVEH exhibits only one resonance frequency at 47.80 Hz. In contrast, the proposed PVEH featuring an additional hollowed trapezoidal cantilever beam possesses two resonance frequencies of 24.20 Hz and 50.90 Hz, wherein 24.20 Hz is the first-order resonant frequency and 50.90 Hz is the second-order resonant frequency. Thus, for the proof-mass-free hollowed trapezoidal cantilever considered in this study, the first- and second-order bending-mode natural frequencies obtained from the FEM model are 24.20 Hz and 50.90 Hz, respectively. Moreover, the first-order peak output voltage of the proposed hollowed trapezoidal cantilever beam PVEH is much higher than that of the conventional solid PVEH. The above results confirm the superior characteristics of the proposed structure.

Output voltages and output powers at different external loads have also been tested and calculated, with the results presented in Figure 2b. In Figure 2b, the voltages represent the peak steady-state values across the external resistive loads at the resonant frequency, rather than the open-circuit voltage shown in Figure 2a. In Figure 2b, the voltages are plotted on the left-hand *y*-axis and represent the peak steady-state values across the external resistive loads at the resonant frequency, whereas the peak power densities are plotted on the right-hand *y*-axis in units of mW/cm^3^. The value close to 55 in Figure 2b therefore corresponds to the maximum power density (55.83 mW/cm^3^) rather than to a voltage amplitude, and the peak loaded voltages read from the left-hand axis are lower than the open-circuit voltage in Figure 2a, in agreement with the expected behavior of a linear piezoelectric harvester. As can be seen, as the external load increased from 100 Ω to 100 kΩ, the output voltages of both PVEHs at their resonance frequencies would initially increase and then tend to stabilize at a certain level. Similarly, the output power increased until it reached a peak value and then decreased. According to the maximum power transfer theorem, the peak output power is achieved when the external load equals the resistance of the piezoelectric cantilever beam. For a sinusoidal voltage across a purely resistive load R with a peak-to-peak amplitude *V_pp_*, the average output power is calculated as $P=V_{pp}^{2}/8R$, and the corresponding peak power density is $P_{eff}=V_{pp}^{2}/8RV_{eff}$, where *V_pp_* represents the peak-to-peak voltage, *R* represents the optimal external load and *V_eff_* represents the effective volume [32].

From Figure 2b, it can be determined that the optimal external loads of the conventional and proposed PVEH are 8.32 kΩ and 7.94 kΩ, respectively. As the effective volume (Veff) of the conventional and proposed PVEHs are calculated to be 0.372 cm^3^ and 0.351 cm^3^, respectively, the peak power densities of these two PVEHs are calculated to be 14.89 mW/cm^3^ and 55.83 mW/cm^3^.

The surface stress distributions of the piezoelectric layer for both PVEHs displayed on the same color scale are shown in Figure 2c and Figure 2d, respectively. These stress cloud maps are obtained under a harmonic base acceleration of A = 1 g (approximately 9.795 m/s^2^), with the excitation frequency set to the first resonant frequency of each structure, i.e., 47.80 Hz for the conventional solid PVEH and 24.20 Hz for the proposed hollowed trapezoidal PVEH. It can be observed that the area of high stress density for the conventional solid PVEH is concentrated near the fixed end, whereas for the proposed PVEH, this area expands towards the free end. Consequently, the design has resulted in an enlargement of the high-stress area, indicating that the proposed PVEH is likely to exhibit superior performance.

## 3. Optimization of the Dimension Parameters

To explore the effect of the hollowed trapezoidal sizes on the characteristics of the proposed PVEH, the output voltages at different frequencies for various trapezoidal dimensions are calculated. Since a hollowed trapezoidal structure with a too large dimension may lead to fracturing of the piezoelectric layer in practical applications, and considering a piezoelectricity layer length of 60 mm, the height of the trapezoid is set to be 40 mm based on our previous work [28]. The widths W1 and W2 of the two bottoms of the trapezoid were selected within a range of 3 mm to 24 mm with a step of 3 mm. Consequently, 56 PVEH models with diverse trapezoidal sizes were initially planned for construction and study.

**Figure 3:**
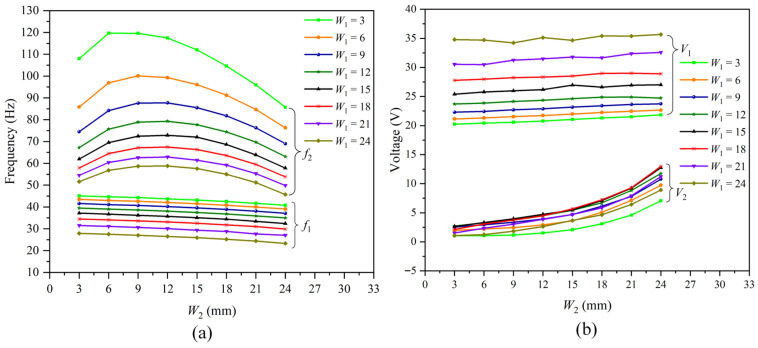
(**a**) The resonance frequencies (f_1_ and f_2_) and peak output voltages (V_1_ and V_2_) of the proposed PVEH are shown as functions of W_1_ at a fixed W_2_, revealing a decrease in f_1_ and f_2_, an increase in V_1_, a non-monotonic V_2_ and optimal performance at W_1_ ≈ 24 mm within the 0–100 Hz range; (**b**) The resonance frequencies and peak output voltages as functions of W_2_ at a fixed W_1_. This shows slight decreases in f_1_, non-monotonous changes in f_2_, modest growth in V_1_ and strong enhancement in V_2_. It also illustrates the influence of the opposite size relationships between W_1_ and W_2_, which are summarised in Table 3. voltages of the proposed PVEH with different widths.

In these models, W1 was fixed to a specific value ranging from 3 mm to 24 mm by a 3 mm step, resulting in, in total, eight certain values comprising 3 mm, 6 mm, 9 mm, 12 mm, 15 mm, 18 mm, 21 mm and 24 mm. For each fixed value of W1, W2 varied from 3 mm to 24 mm by a 3 mm step, wherein the situation when W1 = W2 should be excluded, as this would not constitute a trapezoid. However, in this study, models for the situation W1 = W2 were also constructed and tested, leading to a total of 64 models being constructed. The resonant frequencies and corresponding peak output voltages were investigated and summarized in Figure 3a and Figure 3b, respectively, wherein f1 and f2 represented the first-order and second-order resonance frequencies, V1 and V2 were the corresponding peak output voltages of f1 and f2, respectively.

As illustrated in Figure 3a, for a fixed W2, both f1 and f2 would decrease gradually as W1 increased from 3 mm to 24 mm, while the corresponding V1 would increase accordingly with an approximate fluctuation of 15 V. In contrast, the variation trend of V2 was not as orderly as V1 with a fluctuation of less than 6 V. As an example, when W2 was fixed at 15 mm and W1 increased from 3 mm to 24 mm, f1 decreased from 43.20 Hz to 25.90 Hz, while V1 increased from 21.05 V to 34.67 V. Meanwhile, f2 decreased from 112 Hz to 57.60 Hz and V2 rose from 2.09 V to 6.15 V initially as W1 increased from 3 mm to 15 mm, but then decreased gradually from 6.15 V to 3.68 V as W1 continued to increase from 15 mm to 24 mm. Notably, when W2 = 15 mm, V2 for W1 = 12 mm and W1 = 18 mm were 5.44 V and 5.69 V, respectively, while V2 of W1 = 9 mm and W1 = 21 mm were 4.71 V and 4.72 V, respectively, and the maximum V2 of W2 = 15 mm was obtained in the situation W1 = 15 mm. To achieve a better performance, including higher peak output voltages and lower resonance frequencies within the range of 0–100 Hz, W1 should be set higher than 15 mm. Considering that the amplification of V1 would increase as W1 increased from 15 mm to 24 mm, and that V2 also exhibited favorable values within this range, the optimal value of W1 was set to be 24 mm.

From another perspective, for a fixed value of W1, f1 would decrease slightly as W2 increased from 3 mm to 24 mm, with a corresponding V1 approximately increased slightly accordingly, and f2 would increase initially and then decrease as W1 increased continuously. As an example, when W1 was fixed at 15 mm and W2 increased from 3 mm to 24 mm, f1 would decrease from 37.2 Hz to 32.4 Hz with a corresponding slight increase in V1 from 25.39 V to 27.02 V, excluding the situation W1 = W2 = 15 mm. Meanwhile, f2 exhibited a non-monotonic behavior: it increased from 62 Hz to 72.80 Hz as W2 increased from 3 mm to 12 mm, and then decreased from 72.80 Hz to 57.80 Hz as W2 continued to increase from 12 mm to 24 mm. During this change, V2 increased from 2.65 V to 12.74 V.

To investigate more clearly the impact of the relationship between W1 and W2 on the characteristics of the proposed PVEHs, the results for 12 models with diverse sizes, where both W1 and W2 were greater than or equal to 15 mm, are summarized in Table 3. These 12 models are divided into six groups, with each group containing two models. The two models within a group have the opposite size relationships, for example, if one model has dimensions of W1 = 15 mm and W2 = 18 mm (where W1 < W2), the other model in the same group would have dimensions of W1 = 18 mm and W2 = 15 mm (where W1 > W2).

It can be concluded from Table 3 that, when compared with the situation *W*_1_ < *W*_2_, a smaller *f*_1_ and a larger *V*_1_ are obtained when *W*_1_ > *W*_2_. Furthermore, the magnitude of this variation increases as the difference between *W*_1_ and *W*_2_ widens. To achieve a balance between a potentially lower frequency and a corresponding higher peak output voltage, while preserving the trapezoidal structure, the optimal dimensions of the trapezoidal structure were determined to be *W*_1_ = 24 mm and *W*_2_ = 21 mm.

## 4. Experiment

**Figure 4:**
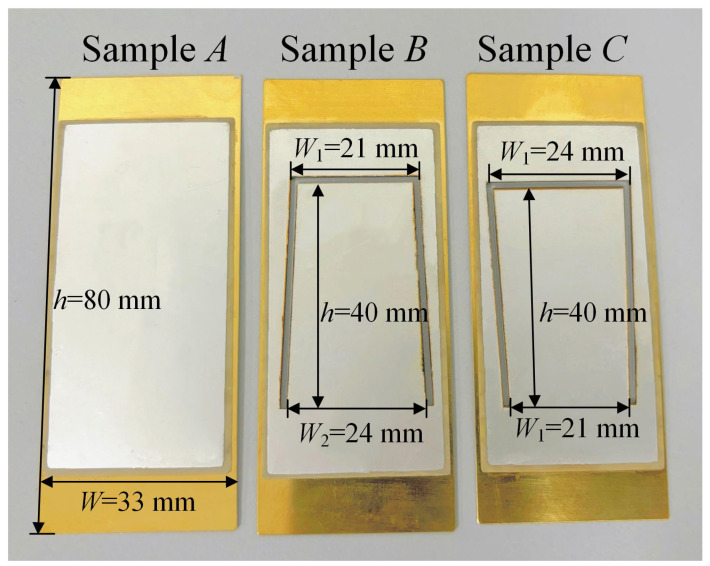
Experimental samples.

Based on the results summarized in Table 3, the two models in group 6 exhibit relatively lower resonant frequencies and higher peak output voltages, with dimensions of W1 = 24 mm and W2 = 21 mm for one model, and W1 = 21 mm and W2 = 24 mm for the other. PVEHs with the above two trapezoidal structures are fabricated and labeled as Sample C and Sample B in Figure 4, respectively. Additionally, a conventional solid PVEH with dimensions of 80 mm × 33 mm is also fabricated and labeled as Sample A for comparative purposes. The piezoelectric layer used in these prototypes is a commercial PZT-5H ceramic, which is a soft PZT material with a Curie temperature of approximately 193 °C, and its basic material properties are listed in Table 1.

The fabrication process of the samples is described as follows: Firstly, the PZT piezoelectric layer and the copper substrate layer were cleaned in an ultrasonic cleaner for 10 min and subsequently placed in an oven at 80 °C to remove any moisture; Next, the surfaces of the PZT piezoelectric layer and copper substrate layer were polished. A layer of conductive silver glue was then screen-printed onto the surface of the copper substrate layer, and the PZT piezoelectric layer was adhered to the copper substrate using the thermocompression bonding technique in a vacuum oven at 200 °C for 3 h. Following this, the sample was gradually cooled down, with the temperature maintained for half an hour for every 30 °C change until it reached room temperature. A 5 μm-thick Ag layer was screen-printed onto the upper surface of the PZT piezoelectric layer to serve as the electrode layer. Subsequently, a high-power laser (1064 nm, RZY-BX-10B, Suzhou Ruizhiyi Laser Technology Co., Ltd., Suzhou, China) was utilized to add the proposed trapezoidal structure to the elastic layer. After the bonding and electrode deposition processes, the assembled samples were polarized along the thickness direction under a DC electric field at an elevated temperature well below the Curie temperature of PZT-5H, so that the high-temperature bonding step at 200 °C was applied to unpolarized plates and did not lead to depolarization of the working devices. The device was cut 4000 times under the conditions of a power of 5 W, a scanning speed of 50 mm/s and a frequency of 20 kHz. Although this laser machining process inevitably induces local heating in a narrow region along the cutting path, the PZT-5H plates are still unpolarized during laser cutting and are polarized only after the machining and electrode deposition steps; together with the fact that the heat-affected zone occupies only a very small fraction of the active area and that the impedance spectra and output voltages of different samples show no obvious degradation, this indicates that the laser-induced local heating does not significantly affect the piezoelectric properties of the fabricated devices.

**Figure 5:**
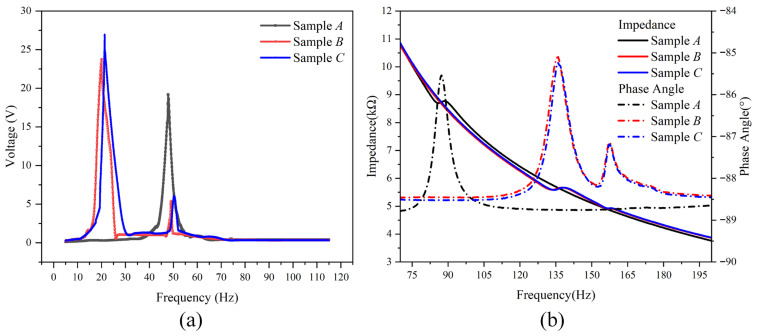
Test results of output voltages (**a**) and impedance and phase angle (**b**) of the fabricated samples.

The fabricated samples were tested using an experimental platform that comprised a dual-channel oscilloscope (UTD2102CEX, Uni-Trend Technology (China) Co., Ltd., Dongguan, China), a function generator (UTG9002C-II, Uni-Trend Technology (China) Co., Ltd., Dongguan, China)), a vibration meter (YE5932, Sinocera Piezotronics, Inc., Yangzhou, China) a power amplifier (YE5872A, Sinocera Piezotronics, Inc., Yangzhou, China)), an impedance analyzer (WK6500P, Wayne Kerr Electronics Ltd., London, UK), and a vibrator (JZK-5, Sinocera Piezotronics, Inc., Yangzhou, China). In particular, the voltage–frequency characteristics shown in Figure 5a are obtained on the shaker under a base acceleration of 1 g with the excitation frequency swept from 0 to 115 Hz, whereas the impedance magnitude and phase angle in Figure 5b are measured in a separate small-signal test using the WK6500P impedance analyzer over a higher-frequency range from 100 Hz to 10 kHz without mechanical excitation.

The test results for the three samples are displayed in Figure 5. As shown in Figure 5a, under an acceleration excitation condition of 1 g (where g = 9.795 m/s^2^) and a sweeping frequency ranging from 0 to 115 Hz with a step of 0.1 Hz, Sample A exhibits a resonance frequency fA of 47.80 Hz and a corresponding peak output voltage VA of 19.19 V. In contrast, both Sample B and Sample C exhibit two resonance frequencies and two corresponding peak output voltages. Specifically, Sample B has resonance frequencies of 20 Hz and 49 Hz with peak output voltages of 23.76 V and 5.39 V, respectively, while Sample C has resonance frequencies of 21.30 Hz and 50.40 Hz with peak output voltages of 26.93 V and 6.08 V, respectively. Therefore, for the fabricated optimal proof-mass-free trapezoidal PVEH (Sample C), the first- and second-order bending-mode resonance frequencies are 21.30 Hz and 50.40 Hz, respectively. Compared to the conventional solid PVEH (Sample A), the first-order resonance frequency f1 of Sample C is reduced by 55.44% with a 40.33% increase in the corresponding first-order peak output voltage (V1). Similarly, the f1 of Sample B is reduced by 58.16% with a 23.81% increase in V1. It is worth noting that, in the FEM optimization results summarized in Table 3 (group 6), the first-order resonant frequency of the model corresponding to Sample C (W1 = 24 mm, W2 = 21 mm) is slightly lower than that of the model corresponding to Sample B (W1 = 21 mm, W2 = 24 mm), whereas in the experiments f1B = 20.0 Hz is slightly lower than f1C = 21.3 Hz. This inversion of the order can be attributed to unavoidable variations in the fabrication process (such as the thickness and elastic properties of the adhesive layer, small tolerances in the laser-machined trapezoidal geometry and residual stresses at the PZT/Cu interface) as well as the idealized boundary conditions and material parameters adopted in the FEM model. Since the first-order resonant frequencies of Samples B and C are very close to each other in both simulation and experiment, these small deviations are sufficient to change their relative order and are within the combined uncertainty of the modelling and measurements.

As illustrated in Figure 5b, the matched loads of Samples A, B and C were measured to be 8.70 kΩ, 5.59 kΩ and 5.58 kΩ, respectively. It should be noted that the impedance curves in Figure 5b are therefore mainly used to determine the equivalent electrical impedance and the matched resistive loads at the resonant frequencies identified from Figure 5a, and they do not display the low-frequency mechanical resonance peaks in the 10–100 Hz band. The maximum output power densities were calculated to be 14.22 mW/cm^3^, 35.86 mW/cm^3^ and 46.29 mW/cm^3^, respectively. For comparison, the simulated first-order resonant frequency and peak output voltage of the optimal trapezoidal PVEH (Sample C), obtained from the FEM model described in Section 2, are 24.20 Hz and 35.28 V with a maximum power density of 55.83 mW/cm^3^, whereas the experimental results exhibit a first-order resonant frequency of 21.30 Hz, a peak output voltage of 26.93 V and a maximum power density of 46.29 mW/cm^3^. These values are summarized in Table 4, and the differences between the simulation and experimental results can be attributed to the idealized boundary conditions and material properties in the simulations, as well as inevitable deviations in the fabrication and test environment.

The results indicate that the trapezoidal structure design effectively enhances the output performance by creating a new second-order resonant frequency with a corresponding second-order peak output voltage, while simultaneously decreasing the value of the first-order resonance frequency with a much higher corresponding first-order peak output voltage. Furthermore, at similar resonant frequencies, Sample C (W1 = 24 mm, W2 = 21 mm) exhibited a higher output voltage compared to Sample B (W1 = 21 mm, W2 = 24 mm), resulting in a superior performance of Sample C.

To demonstrate the capability of harvesting vibration energy from the environment and utilizing this energy to power devices, a simple application was implemented through an energy harvesting circuit. Initially, an ideal FBR circuit was adopted for the proposed PVEH; however, the output voltage was only 3.08 V at an electronic load of 1000 Ω. Furthermore, since the capacitance inside the PVEH needed to be charged during each cycle, charge loss would occur. In this study, a P-SSHI-QVR circuit is proposed using the charge-flipping technique to harvest alternating current electrical energy from the proposed optimal trapezoidal cantilever beam. The logic for controlling the switches in this circuit is relatively simple and possesses the ability to rectify and amplify weak electric signals.

**Figure 6:**
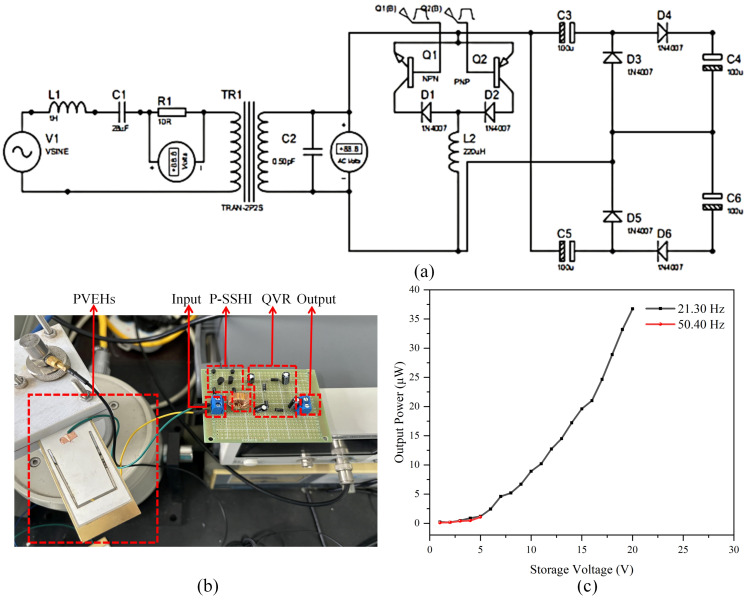
Schematic diagram (**a**), physical diagram (**b**), and output power results of the P-SSHI-QVR circuit (**c**).

The schematic diagram of the P-SSHI-QVR circuit is shown in Figure 6a. An LRC oscillation circuit is used to simulate the AC signal generated by the resonance of the trapezoidal cantilever beam. The proposed P-SSHI-QVR circuit comprises Q1 and Q2 switching tubes, Schottky diodes, inductors, and other components. The physical diagram of the P-SSHI-QVR circuit is presented in Figure 6b, including an input port, a P-SSHI charge flipping link, a QVR rectification link, and an output port. Figure 6c depicts the storage voltage and output power results of the P-SSHI-QVR circuit under different resonant peaks of the PVEHs. The measurement results demonstrate that the output power increases as the storage voltage rises, regardless of whether it is stored at a high voltage corresponding to the first-order resonant frequency of 21.30 Hz or at a low voltage corresponding to the second-order resonant frequency of 50.40 Hz. Therefore, the circuit is capable of storing electrical energy at both resonance peaks. However, due to the inability to increase the corresponding storage voltage above 5.68 V at 50.40 Hz, the image persistence at 50.40 Hz is not as long as that at 21.30 Hz. The maximum stored voltage reaches 20.49 V at 21.30 Hz with a maximum stored power of 36.89 μW, compared to only 2.97 μW at 50.40 Hz.

## 5. Conclusions

In this study, the structure and characteristics of a trapezoidal hollowed PVEH equipped with a P-SSHI-QVR circuit for low-frequency applications are thoroughly investigated and optimized through both simulation and experimental measurements. It was discovered that when the bottom width of the trapezoidal structure near the fixed end (W1) was larger than that near the free end (W2), the peak output voltage would increase. As a result, an optimal trapezoidal PVEH with dimensions of W1 = 24 mm and W2 = 21 mm was obtained.

Compared with the traditional cantilever beam PVEH, the first-order resonant frequency of the proposed PVEH was reduced by 55.44% with corresponding increases in the first-order peak output voltage and maximum power density by 40.33% and 225.53%, respectively. Furthermore, the P-SSHI-QVR circuit was proposed to harvest the AC electrical energy from the optimal PVEH. The maximum stored voltage was 20.49 V at a first-order resonant frequency of 21.30 Hz and 5.68 V at a second-order resonant frequency of 50.40 Hz, with corresponding maximum stored powers of 36.89 μW and 2.97 μW, respectively. In addition, for ultra-low-frequency vibration environments where the operating frequency is significantly lower than 20 Hz, the proposed trapezoidal cantilever beam can be further integrated with existing frequency up-conversion schemes, such as impact- or plucking-type mechanisms, to extend the effective operating range. This combination will be investigated in our future work.

## 6. Patents

Feng B., “Micro energy harvesting system,” Chinese invention patent, Application No. CN202311682148.3, Publication No. CN117811410A, under substantive examination.

## Figures and Tables

**Table 1:** Material properties of the different structures.

Structure	Electrode Layer	Piezoelectric Layer	Substrate Layer
Material	Ag	PZT-5H	Copper
Density (kg/m^3^)	10,500	7500	8780
Yang’s modulus (10^10^ N/m^2^)	83	5.6	11.2
Poisson’s ratio	0.37	0.36	0.35

**Table 2:** Dimension parameters of the different layers.

	Electrode Layer	Piezoelectric Layer	Substrate Layer
Length (mm)	60	60	80
Width (mm)	31	31	33
Thickness (mm)	-	0.2	0.2

**Table 3:** Comparison of the peak voltage outputs of the proposed PVEH with different widths.

Group	*W*_1_	*W*_2_	*f*_1_	*V*_1_	*f*_2_	*V*_2_
1	15	18	34.4	26.62	68.6	7.11
	18	15	32.5	28.53	66.3	5.69
2	15	21	33.4	26.95	63.9	9.23
	21	15	29.3	31.78	61.4	4.72
3	15	24	32.4	27.02	57.8	12.74
	24	15	25.9	34.66	57.6	3.68
4	18	21	31.0	28.99	59.5	9.30
	21	18	28.8	31.65	59.2	6.54
5	18	24	29.9	28.91	53.8	12.99
	24	18	25.2	35.42	55.0	5.09
6	21	24	27.0	32.57	49.8	12.56
	24	21	24.4	35.39	51.2	7.53

**Table 4:** Comparison between simulated and experimental results of the optimal trapezoidal PVEH (Sample C).

Method	f1 (Hz)	V1 (V)	Maximum Power Density (mW/cm^3^)
Simulation	24.20	35.28	55.83
Experiment	21.30	26.93	46.29

## Data Availability

The data presented in this study, including simulation results and experimental measurements of the proposed PVEH and P-SSHI-QVR circuit, are available from the corresponding author upon reasonable request.
